# Innate Immune DNA Sensing of Flaviviruses

**DOI:** 10.3390/v12090979

**Published:** 2020-09-03

**Authors:** Tongtong Zhu, Ana Fernandez-Sesma

**Affiliations:** 1 Department of Microbiology, Icahn School of Medicine at Mount Sinai, New York, NY 10029, USA; tongtong.zhu@icahn.mssm.edu; 2 Graduate School of Biomedical Sciences, Icahn School of Medicine at Mount Sinai, New York, NY 10029, USA

**Keywords:** flavivirus, innate immunity, pathogen sensing, viral antagonism, DNA sensing

## Abstract

Flaviviruses are arthropod-borne RNA viruses that have been used extensively to study host antiviral responses. Often selected just to represent standard single-stranded positive-sense RNA viruses in early studies, the *Flavivirus* genus over time has taught us how truly unique it is in its remarkable ability to target not just the RNA sensory pathways but also the cytosolic DNA sensing system for its successful replication inside the host cell. This review summarizes the main developments on the unexpected antagonistic strategies utilized by different flaviviruses, with RNA genomes, against the host cyclic GAMP synthase (cGAS)/stimulator of interferon genes (STING) cytosolic DNA sensing pathway in mammalian systems. On the basis of the recent advancements on this topic, we hypothesize that the mechanisms of viral sensing and innate immunity are much more fluid than what we had anticipated, and both viral and host factors will continue to be found as important factors contributing to the host innate immune system in the future.

## 1. Introduction

**Figure 1:**
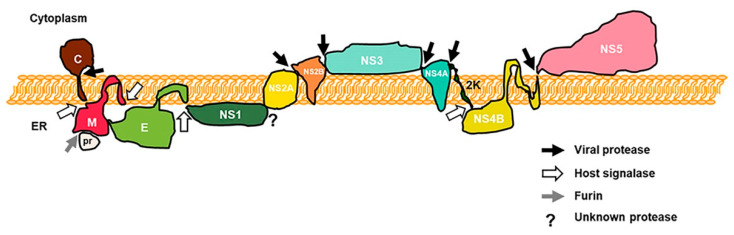
Schematic of a flavivirus polyprotein in the endoplasmic reticulum (ER). Capsid (C), prM, and envelop (E) proteins facilitate viral entry and maturation, while the other seven nonstructural (NS1, NS2A, NS2B, NS3, NS4A, NS4B, and NS5) proteins are responsible for viral replication, viral particle assembly, and maturation. Viral and host proteases cleavage sites are indicated by arrows. NS4A C-terminal transmembrane domain 2K is also shown (adapted from J.R Rodriguez-Madoz, unpublished).

Flaviviruses are a group of important emerging and re-emerging pathogens that are mostly arthropod-borne. Several members of the genus, such as dengue virus (DENV), Zika virus (ZIKV), West Nile virus (WNV), yellow fever virus (YFV), and Japanese encephalitis virus (JEV), are major international public health concerns [1,2,3,4,5,6]. Flaviviruses’ viral particles consist of three structural proteins, a lipid envelope, and a positive-sense genomic RNA about 11 kb in length with structurally relevant non-coding regions on both the 3′ and 5′ ends. Their genomes encode a single polyprotein that is processed during and after translation by both host and viral proteases into ten individual proteins (Figure 1) [7].

Out of the ten, three are structural proteins (capsid, prM, and envelope) that facilitate viral entry and maturation, and seven are nonstructural proteins (NS1, NS2A, NS2B, NS3, NS4A, NS4B, and NS5) that are responsible for viral replication, viral particle assembly, and maturation [8]. Because of their limited number of proteins, flaviviruses must rely on clever strategies to antagonize host innate and adaptive immune systems to replicate successfully [3,9]. Stimulator of interferon genes (STING), an important adaptor of the human innate immune DNA sensory pathway, has been reported to be targeted for degradation by DENV, an RNA virus, to inhibit innate immune responses [10,11]. These observations led the way for additional studies on flaviviruses’ direct regulation on this host cytosolic DNA sensing system, which will be the focus of this review.

## 2. Innate Immune System

### 2.1. Overview

The clearing of an infection caused by pathogens (such as viruses, bacteria, fungi, or parasites) in humans is usually the result of a close collaboration between two major types of immune responses, namely the innate and adaptive immunity. During such an infection, a local innate immune response serves to quickly detect and contain the infection and then, through the help of a heterogeneous group of immune cells (such as Langerhans cells and other dendritic cells, macrophages, B cells, T cells, and others), activate an adaptive immune response that would further control and eventually eliminate the infection [12]. Given that innate immunity plays an essential role in the host’s defense against pathogen invasions, it is perhaps not surprising that many viruses have successfully evolved strategies to regulate or even antagonize the innate immune system during their early stages of infection [13,14]. Robust innate immune responses must rely on an extensive and sensitive network of surveillance proteins, or pattern recognition receptors (PRRs), to detect intruders exhibiting pathogen-associated molecular patterns (PAMPs) and mount proinflammatory and antimicrobial responses. In order to counter them, viruses have evolved ways to avoid detection and make sure they can “fly under the radar” and establish infection in the host [15,16,17].

The list for the currently known innate immune PRRs is extensive and has been steadily expanding in the past three decades since the discovery of the first Toll-like receptors (TLRs) by Hoffmann in 1996 after Janeway’s earlier prediction [18]. Now, the field has advanced tremendously beyond the TLR family, adding new sensors like the nucleotide-binding oligomerization domain-like receptor (NLR) family and its subfamilies (such as NLRP), RIG-I-like receptor (RLR) family, 2′-5′ oligoadenylate synthase (OAS) family, DEAD box polypeptide 41 (DDX41), stimulator of interferon genes (STING), cyclic GAMP synthase (cGAS), IFN-γ-inducible protein 16 (IFI16), and others to the long roster of molecular sentinels watching the host cellular space [19,20,21,22,23,24,25,26]. These receptors and their respective signaling pathways have been actively studied and described based on the pathogen components that they detect (foreign lipids, proteins, or nucleic acid structures or sequences) along with their proposed cellular localizations [27,28].

### 2.2. Viral Nucleic Acid Sensing

Because in most types of cells, active replication of viruses results in an accumulation of intracellular nucleic acids, both host cytosolic DNA and RNA sensors and their respective pathways have been areas of active investigation in the innate immunity field after their discoveries [29,30]. These nucleic acid sensors are capable of detecting their respective ligands, initiating signaling cascades through interaction with adaptor proteins, triggering expression of type I interferons (IFN-I), and mounting an innate immune response by the release of cellular proinflammatory cytokines and chemokines, all of which are essential in defending against viral infection [14,17,31,32,33,34]. Additionally, in response to IFN-I production, there is an induction of IFN stimulated genes (ISGs) in the same cell and bystander cells that results in an inhibition of viral replication in those cells, which is termed the antiviral state [35]. More recently, the sensors’ ability (or inability) to differentiate self versus non-self elements in the cellular space has caught the attention of a wide number of groups that are actively investigating their roles in mutagenesis and autoimmune diseases [36,37,38,39].

### 2.3. Innate Immune DNA Sensors

Host DNA sensors known to play important innate immune functions include TLR9, IFI16, absent in melanoma 2 (AIM2), DDX41, cGAS, and others. Detailed reviews of the current list of DNA sensors have been published extensively by different groups [14,34,40,41]. In this review, we will highlight some key players that have been intensely studied in the field of viral innate immunity.

It is worth pointing out that TLR9 mainly serves as the endosomal DNA sensor, while others are known to play parts in detection of DNA in the cytosolic space. After activation by unmethylated CpG-DNA in the endolysosomal system, TLR9 responds by either engaging interferon regulatory factors (IRFs) to induce IFN-I production or recruiting nuclear factor NF-κB, which leads to the production of proinflammatory cytokines [42,43]. IFI16, a member of the IFN-inducible Pyrin and HIN domain (PYHIN) gene family, was first characterized as a nuclear protein as it harbors a nuclear localization signal (NLS) in the N-terminus [44]. Since then, reports have surfaced finding IFI16 in both nuclear and cytoplasm compartments across different cell types [45]. Although more studies are needed to understand the exact mechanisms governing its subcellular localization, IFI16 is known to induce the activation of inflammasomes as well as the IFN-I response via a STING-dependent manner after detecting dsDNA, or other nucleotide structures [46,47,48]. More on the STING activation pathway will be discussed in the next section. AIM2, also a member of the PYHIN family, was characterized as the activator of the inflammasome and pyroptosome responses after cytoplasmic DNA detection [49]. The assembly of inflammasomes leads to the activation of downstream inflammatory caspases, which in turn can mediate important proinflammatory cytokine interleukin-l beta (IL-lβ) and IL-18 production and pyroptosis, a proinflammatory type of cell death [50,51]. DDX41 was first identified as an intracellular DNA sensor in dendritic cells during a siRNA screen among 59 members in the DEAD box family [52]. It can directly bind DNA and STING and activate IFN-I production through a STING-dependent pathway [24]. Z-DNA binding protein 1 (ZBP1, also known as DAI) is a cytosolic DNA sensor that has been implicated in RNA virus sensing (more on ZBP1 and *Flavivirus* will be discussed later in this review) [53,54,55]. The current list of proposed cytosolic DNA sensors will eventually include proteins that are previously known to have been involved in DNA damage response pathways, transcription, inflammasome assemblies, and other cellular functions [56,57,58,59,60]. Although there are a few publications on some of these DNA sensors and their proposed roles in controlling *Flavivirus* infection in different biological systems, there is yet to be any evidence of antagonism of these pathways by flaviviruses, other than the cGAS/STING pathway [61,62,63,64].

### 2.4. cGAS/STING Pathway

Among all the known innate immune sensors that recognize DNA, the cGAS/STING pathway has served as the principal human cytosolic DNA sensing system (RIG-I/MAVS pathway being the main cytosolic RNA sensing counterpart) ever since their respective discoveries in 2013 and 2008 [14,23,65,66,67]. It is also the host DNA sensing pathway that has been shown to be most implicated during flaviviruses replication and studied in that context. This innate immune sensory system surveys the host cytosolic space for double-stranded DNA and RNA/DNA hybrids regardless of the specific sequence, whose presence usually indicates a pathogen infection or DNA damage inside the nucleus [37,68,69,70]. Activated cGAS produces secondary messenger 2′3′-cGAMP through dimerization that can diffuse across neighboring cells and bind and activate downstream adaptor STING [71,72].

STING (also known as MITA, MPYS, ERIS, or TMEM173) is a transmembrane innate immune signaling adaptor that relays alert signals from activated cGAS to IFN-I and NF-κB pathways in order to mount an inflammatory response against the detected danger signal [31,32]. At resting state, STING associates with stromal interaction molecule 1 (STIM1) until it interacts with cGAMP (produced by cGAS or endogenously by bacteria), which disrupts this interaction and induces STING to translocate from the ER to the ER-Golgi intermediate compartment (ERGIC) to recruit and activate TANK-binding kinase 1 (TBK1), followed by the recruitment and activation of IRF3. The dimerized IRF3 can then translocate into the nucleus and promote IFN-I expression, which leads to a global upregulation of a set of ISGs critical for mounting an effective innate immune defense against sources of danger [72,73,74]. Aside from cGAS, STING seems to also play a part in downstream signaling of other DNA sensors (such as DDX41 and IFI16) [20,24,56,75].

In addition to its canonical role played in the cytosolic DNA sensory pathway, STING also was reported to interact with mitochondrial antiviral signaling protein (MAVS, also known as VISA, IPS-1, or Cardif) both in the presence and absence of Sendai virus (SeV) infection and with RIG-I during SeV infection [65]. In another report, endogenous STING was shown to associate with RIG-I in normal human umbilical vein endothelial cells (HUVECs) either directly or indirectly as a complex [67]. Other groups have since reported more evidence on this interaction [76,77]. However, the exact role STING plays in cytosolic RNA sensing and anti-RNA virus defense is still unclear and needs further exploration [78].

As we learn more about the mechanisms behind cGAS/STING sensing and activation, it has become increasingly clear that this pathway plays an essential role in host innate immunity, especially in regulating antiviral responses [79]. This is perhaps why many viruses, mostly DNA viruses, are found to have evolved strategies to target this pathway for disruption. An excellent review of different ways DNA viruses sabotage this cellular information relay highway was published by Ahn and Barber [80]. For more on the history of the cGAS/STING signaling pathway’s discovery and what we currently know about its various broader cellular functions, readers are suggested to consult this detailed review [81]. For the remainder of this review, the focus will be on how one specific genus of RNA virus, namely flaviviruses, has helped widen our understanding of the scope of cGAS/STING innate immune sensing in the context of antiviral defense. We will focus on several members of this genus and their different strategies in regulating cGAS or STING to illustrate the current knowledge on this topic (summarized in Table 1).

## 3. Dengue Virus

### 3.1. Overview

Dengue virus (DENV) is transmitted by *Aedes aegypti* mosquitoes and is the most prevalent arthropod-borne virus in the world, infecting around 400 million people annually [90]. Roughly a quarter of global DENV infections present as non-specific febrile illness (previously called dengue fever (DF)), of which a small but significant percentage results in more severe infections presenting with hemorrhage or circulatory failure (previously called dengue hemorrhagic fever (DHF) or dengue shock syndrome (DSS), respectively) [91]. DENV exists and circulates as four distinct serotypes (DENV 1–4). Previous exposure to any of the four serotypes can lead to a more severe clinical manifestation of a second infection by another serotype [92,93,94]. Since the first isolation of DENV in 1943, we have seen a rapid expansion of the co-circulation of DENV serotypes around the world, accompanied by an increase in the global incidence rate [95].

### 3.2. Regulation of cGAS and STING by DENV

Out of all the flaviviruses, the antagonism of DENV against the cGAS/STING pathway was reported the earliest. After being the first to discover that DENV2 infection makes monocyte-derived dendritic cells (MDDCs) unable to prime T cells because of a lack of IFN-I production, our group went on searching for the mechanism behind this virus-induced immune modulation [96,97]. We found that the decreased IFN-I production is dependent on a catalytically active NS2B3 protease complex, consisting of the nonstructural proteins NS3 and its NS2B cofactor of DENV [98]. At that time, only ways for DENV to manipulate IFN-I downstream signaling pathway had been reported [99,100,101]. The mechanism behind how DENV avoids triggering IFN-I induction, however, remained elusive.

Later, we (as well as the Yu group) reported that DENV protease complex NS2B3 interacts with and cleaves the innate immune adaptor molecule STING, thus inhibiting IFN-I induction in infected human cells [10,11]. In that study, we initially looked for host proteins containing a putative DENV protease cleavage site through a bioinformatic search among players of the IFN-I induction pathway [102]. Out of the list of candidates that we had subjected to testing, only human STING (hSTING) was susceptible to cleavage by NS2B3. Using both the wildtype (WT) DENV2 NS2B3 and the proteolytically inactive S135A NS2B3 constructs, we observed the cleavage of hSTING, but not murine STING (mSTING) in an ectopically expressed system and that mSTING strongly restricts the replication of DENV in mouse cells. We proposed that this is at least partially owing to the difference in amino acid sequence around the putative cleavage site between hSTING and mSTING. After validating and confirming the results in a primary cell system (MDDCs) that are targets for DENV infection in vivo, we reported that the proteolytic activity of the DENV protease complex is crucial for the degradation of STING, which leads directly to the antagonization of IFN-I production in DENV infected cells. Ours was one of the first reports showing STING is targeted by a viral protein (paradoxically of an RNA virus) for cleavage and degradation (reviewed in [32]).

Although we have also performed infections using different DENV serotypes (DENV-2 16681 strain, DENV-3 PR-6 strain, and DENV-4 H-241 strain) and observed similar results, the data were not included in that publication. Later, in 2015, the Mackow team included the finding that DENV4 NS2B3 similarly cleaves STING using an overexpression system in their publication on DENV inhibition of RIG-I/MAVS signaling [83]. This suggested conservation of innate immune regulatory mechanisms among DENV serotypes, which was confirmed later by another study testing NS2B3 constructs from all four serotypes in their ability to cleave STING [84]. In this study done by the Sawyer group, the authors proposed position R78 and G79 on STING to be an interspecies cleavage determinant for the DENV protease complex. However, in their attempt to try to validate their prediction using STING constructs from naked mole rat, desert woodrat, and chinchilla, the results were not conclusive.

A study would soon be published and demonstrate more evidence to support the newly proposed cleavage site [85]. While using an overexpression model, the researchers noticed that, when the previously proposed cleavage site R95/G96 was replaced with those of the mouse sequence, hSTING was cleavable by DENV2 NS2B3 [10,11]. Moreover, when the newly proposed cleavage site R78/G79 was mutated, DENV NS2B3 would seem to fail to cleave these versions of recombinant hSTING (the G79D mutant was cleaved less), as expected. The caveat here which the authors noted as well was that, even when replaced with a stretch of human sequence that included the proposed new cleavage site, the recombinant mSTING was still not cleavable by WT ZIKV NS2B3, which is the main target of investigation for this study. Because the experiment overexpressing DENV NS2B3 and this minimally humanized mSTING was not done, we do not know if DENV protease can cleave a recombinant mSTING with a human cleavage site. It seems additional amino acid sequences, conformational, or even host or viral chaperon cleavage determinants, may still exist and are waiting to be discovered [8]. More on this study will be discussed in the next section.

Lastly, an ER protein SCAP was proposed by the Wang group to be the host’s countermeasure to the DENV protease complex’s disruptive ability [103]. Using an overexpression system, they reported that, by binding to NS2B, SCAP renders the K27-linked ubiquitination of NS3 ineffective, thus impairing the DENV NS2B3’s ability to cleave STING. It would be interesting to see if the endogenous SCAP level in a primary cell system can be a predictor for DENV infectivity both in vivo and in vitro.

Our team continued investigating DENV’s strategies in modulating and evading the host cGAS/STING pathway, and we found that cGAS was also degraded during DENV infection [82]. Combined with the previous knowledge that part of the cGAS/STING pathway is a target for DENV antagonism and the observation that MDDCs infected with DENV demonstrated a reduction in their ability to induce IFN-I and ISG15 mRNA, we proceeded to study if cGAS was impacted during infection [10,11]. In that study, we described that the DENV protease cofactor NS2B targets cGAS for degradation through an autophagy–lysosome-dependent mechanism, resulting in inhibition of the cytosolic DNA sensing pathway in DENV infected cells (including human MDDCs) [96]. Through biochemical and functional analysis in cell lines and MDDCs, we reported a significant inhibitory effect exerted by the DENV NS2B3 complex on the cGAS/STING pathway and that the pathway displays an inhibitory effect on DENV replication if left intact [82]. This was the first report showing cGAS is targeted by a viral protein (again, paradoxically, of an RNA virus) for degradation (reviewed in [104]).

The most recent development in this area comes from the investigation on the roles played by the profile of host STING haplotype and the state of coinfection of bystander cells during DENV infection by the Yu group [86]. Using data from the National Center for Biotechnology Information (NCBI) 1000 Genomes Project, their team discovered that the four missense variations at residues 71, 230, 232, and 293 of STING and their respective combinations are responsible for creating the three most common haplotypes (RGRR, HARQ, and RGHR) in the human population. In addition, it was shown that the DENV2 protease cleaves hSTING differently depending on the haplotype; whereas the HARQ type is more susceptible to cleaving, the RGHR type demonstrates resilience. Furthermore, they reported that, in the presence of 2′3′-cGAMP (directly applied or produced by the activation of cGAS in neighboring cells), the haplotype HARQ is more susceptible to cleaving by DENV2 NS2B3. The authors proposed that the conformational changes experienced by STING in the presence of its activator may contribute to the enhanced cleaving by the DENV protease complex. Nonetheless, this observation has shed important light on host factors that may play a role in further modulating the interaction of DENV NS2B3 and hSTING during infection. Future studies are warranted to better understand the molecular events that took place during the cleaving and degradation of STING by DENV protease.

Beyond cGAS/STING, researchers from the Ho group have also reported that the TLR9 signaling pathway is activated during DENV infection in human dendritic cells [62]. Using immunoprecipitation and qPCR assays, they have shown that TLR9 can detect leaked mitochondria DNA (mtDNA) during DENV infection. If confirmed, it would be interesting to test and see whether DENV can disrupt the activation of TLR9 in the same fashion as it antagonizes cGAS/STING.

## 4. Zika Virus

### 4.1. Overview

The Zika virus (ZIKV) is a member of the *Flavivirus* genus and transmitted by *Aedes aegypti* mosquitoes, and it has recently received much attention for being the causative agent behind the 2015 epidemic in South America [105]. First isolated in 1947 from a rhesus monkey, ZIKV infection was associated with only mild illness before the large French Polynesian outbreak between 2013 and 2014, when neurological complications, including Guillain–Barré syndrome, were first observed in patients [105,106,107]. After it was widely considered to have caused severe birth defects like congenital microcephaly and intracranial calcifications following in utero exposure, the World Health Organization (WHO) declared ZIKV a Public Health Emergency of International Concern in early 2016 [108,109,110,111]. Although herd immunity and other factors have largely blunted ZIKV’s epidemic potential and the number of incidences worldwide has decreased significantly over the past several years, it still harbors the potential to re-emerge again without any effective vaccines or antivirals currently being available [112,113].

### 4.2. Regulation of cGAS and STING by ZIKV

As early as 2015, during an investigation into the positive selection targets in ZIKV and related flaviviruses, two positively selected sites (M87 and H88) were identified in the N-terminal region of ZIKV NS4B, which are believed to be involved in host protein (e.g., STING) binding [114]. The authors proposed that this is the result of adapting to better modulate innate immune sensors during viral evolution. At this point, no interaction between ZIKV nonstructural proteins and STING was known. In 2017, for the purpose of finding human cGAS/STING pathway agonists, a HepAD38-derived reporter cell-based high throughput screening assay was developed [115]. Their hit compound, a dispiro diketopiperzine (DSDP), was found to activate hSTING and induce IFN-dominant cytokine responses, but did not activate mSTING. In a study using multiple flaviviruses (DENV, ZIKV, and YFV) to infect DSDP-activated THP cells, the authors observed a dose-dependent reduction in both viral RNA level and titer release for all viruses. This is the first clear indication that STING is a restriction factor for ZIKV replication in human cells.

In 2018, the Ploss team tested ZIKV’s ability to infect a diverse range of mammalian cells in order to determine its host tropism [85]. They observed that the human, great ape, and Old and New World monkey cells are susceptible to ZIKV infection, but the rodent cells are not. From a variety of cell-intrinsic defense mechanisms that could potentially explain ZIKV tropism in different mammalian cells, they tested the cGAS/STING pathway, as it was known to be an important pathway targeted by DENV and also is not universally conserved among mammals [10,11,84,116]. It was discovered that ZIKV NS2B3 targets human, but not murine, STING for proteolytic degradation with the proposed cleavage site at R78/G79 in the cytoplasmic loop of human hSTING in both an overexpression system and human fibroblasts. As previously discussed in this article, the authors also reported that the recombinant hSTING was cleavable by the ZIKV protease construct when the previously proposed cleavage site R95/G96 was replaced with those of the mouse sequence [10,11]. Nonetheless, similar to what has been observed with the previously proposed site, when the new hSTING cleavage site was inserted into mSTING, it would remain uncleavable by the viral protease. Another point of interest is that, in the same study, the mutant chimpanzee STING (W78R) that harbored a human cleavage site was cleavable by ZIKV NS2B3, but this was not true for mutant rhesus macaque STING (D79G), which also had a human cleavage site. Altogether, future studies are needed to tease apart the interaction mechanism of *Flavivirus* protease complexes with cGAS/STING pathway components more closely, ideally with the findings confirmed in a primary cell system. Furthermore, although it was observed through loss-of-function assays that mSTING knockout (KO) cells exhibit increased permissiveness to ZIKV, Goldenticket mice that harbor a missense mutation in exon 6 of the *Sting* gene (*Tmem*173^Gt^) did not show elevated susceptibility to ZIKV infection [117]. Therefore, the exact mechanism that explains the interspecies variation to ZIKV infection also remains to be elucidated.

Later in the same year, it was reported by the Cui group that ZIKV can target cGAS for degradation via the enhanced stabilization of caspase-1 by nonstructural protein NS1 during infection [87]. Unlike its flavivirus cousin DENV, ZIKV has adopted a more indirect strategy in antagonizing cGAS. Whereas DENV viral protein NS2B interacts directly with cGAS and facilitates its degradation through an autophagy–lysosome-dependent mechanism, ZIKV NS1 is found to actively recruit deubiquitinase (DUB) USP8 and remove the K11-linked ubiquitin chains at the lysine 134 position of caspase-1, thus increasing the stability of caspase-1 and the cleavage of cGAS during infection [82,87]. Care was also taken in this report to examine the connection between the degradation of cGAS and the attenuation of IFN-I response observed during ZIKV infection using cGAS KO assays in both cell lines and PBMCs from healthy donors. In those cases, the absence of cGAS led to enhanced ZIKV replication and impaired IFN-β production [87]. Later, when cGAS KO THP-1 cells were reconstituted with a mutant form of cGAS that was not cleavable by caspase-1, a stronger antiviral response was observed along with a decreased level of ZIKV replication.

Beyond the mammalian systems, researchers have recently discovered that *Drosophila* STING (dSTING) protects against ZIKV infection by inducing autophagy in the brains of adult flies [118,119,120]. It was proposed that dSTING is an essential component of the innate antiviral defenses in flies in the same way that hSTING is for humans [119]. Although hSTING and dSTING are indeed evolutionarily distant, they all seem to share deep conservation in their ability to induce autophagy in both mammals and insects, with the domain required for NF-kB and IRF3 activation in hSTING only to emerge during vertebrate evolution [121,122,123,124]. So far, no studies on hSTING-induced autophagy during ZIKV infection in mammalian cells have been published. Nevertheless, we are confident that more data will be forthcoming, linking hSTING closer to RNA virus defense.

Beyond cGAS/STING, using a murine model, the Oberst group has reported that the activation of ZBP1 and the downstream receptor interacting protein kinases 1 (RIPK1) and RIPK3 during infection is important in controlling ZIKV pathogenesis in the central nervous system (CNS) [63]. More studies could verify this finding in humans and discover potential virus–host interactions between ZIKV viral proteins and the ZBP1 sensing pathway.

## 5. West Nile Virus

### 5.1. Overview

WNV, transmitted by *Culex* mosquitoes, was first isolated in 1937 from the West Nile district of Uganda and has since caused many epidemics in both animals and humans in all continents (except Antarctica) [2,5]. WNV is known for its broad tropism and can replicate in many different cell types and species (humans, horses, birds, and other wildlife species)—especially notable is the damage it can cause to the CNS [125]. West Nile fever develops in approximately one-fourth of the infected patients, with a wide range of clinical manifestations. Although WNV infection can be contained outside the CNS, it is still capable of evading the innate immune responses and invade the CNS to cause encephalitis, encephalomyelitis, or even death in some patient populations [5,126,127,128,129,130].

### 5.2. Regulation of STING by WNV

In a search for new regulators of IFN-I, the Fikrig group discovered that Goldenticket mice, which are STING deficient, are more susceptible to WNV infection than the WT animals [117,131]. To further explore STING-related innate immune responses, their infection interaction screen (using WNV as the infecting agent) revealed that E74 like ETS transcription factor 4 (ELF4) protein associates with hSTING during infection. The study showed that, after recruitment by STING, ELF4 interacts with and is activated by MAVS/TBK1 and then translocates into the nucleus to induce IFN-I production. This suggests that STING, along with ELF4 and the rest of the important components of the pathway (e.g., IRFs and NF-κB), could be a restricting factor for WNV replication.

Then, in that same article published by the Ploss group, WNV protease was cloned and expressed with both hSTING and mSTING in an overexpression study [85]. This would be the first published evidence that the WNV NS2B3 protease complex is capable of cleaving hSTING. It also suggested that WNV NS2B3 cleaves hSTING, but not mSTING, at the proposed R78 and G79 position, similar to DENV, as the R78Q/W and G79D mutants did not seem cleavable.

Most recently, the Gale group studied the role of STING played in controlling WNV infection using a murine model of infection [130]. First, they validated the observation that STING KO mice have higher morbidity and mortality rates when compared with WT mice during WNV infection [131]. Then, it was suggested that (1) mSTING does not play a protective role in neurons and CNS during WNV infection, (2) the lack of mSTING seems to induce an even higher level of innate immune response signals, and (3) mSTING is not even activated during WNV infection in mice. This would suggest a counterintuitively proviral role for mSTING during WNV infection had the study concluded here. However, this study eventually shows, by cytokine and chemokine profiling, flow cytometry, and histological analyses, that mSTING noncanonically plays a vital role in the proper programming of the T cell response during WNV infection and in the maintenance of a proper balance of immunopathogenic and immunoprotective adaptive immune response to WNV in the CNS. This report provided additional insights on interspecies differences regarding the anti-flaviviral functions of murine and human STING.

Although no published studies have covered the relationship between cGAS and WNV yet, there is a recent report on the important role played by ZBP1 during WNV infection in mice [64]. Adopting similar strategies as the Gale group, Rothan et al. used ZBP1 KO murine model to investigate the role this innate immune nucleic acid sensor played in restricting WNV and ZIKV infection and reported that ZBP1 suppresses their replication in mouse cells. It would be interesting to know if there is any cross-talk between ZBP1 and the cGAS/STING pathway in this context.

## 6. Other Flaviviruses

### 6.1. Yellow Fever Virus

YFV is also transmitted by *Aedes aegypti* mosquitoes and was first isolated from the blood of a patient in Ghana in 1927, and an effective vaccine (17D strain) was subsequently made available in 1937 [7]. Despite this, YFV infections have remained a threat to global health, with outbreaks expanding into new areas that put large populations in South America and Africa at risk [1,132]. YFV is currently endemic in over 45 countries globally and causing around 200,000 severe cases and up to 60,000 deaths every year according to a WHO estimation [132].

As early as 2009, YFV has become known as having the ability to inhibit STING’s ability to induce IFN-I [19]. Simply an attempt to test if the known homology between *Flavivirus* NS4B and hSTING indicated a potential interaction; it was shown that the STING-induced IFN-I signaling decreases with an increasing amount of transfected YFV NS4B in cell lines. However, the exact mechanism of this action is not known. In 2011, a screen was conducted by the Rice group using over 380 ISGs to test their ability in antagonizing the replication of a few important viruses, with YFV being the only flavivirus selected for the screen [133]. In this report, cGAS’s ability to inhibit flavivirus replication was suggested, albeit at a modest degree compared with some other ISGs surveyed. In a 2014 follow-up paper, cGAS was highlighted as a DNA sensor whose expression broadly inhibits several RNA viruses, including YFV [134].

Nevertheless, the relationship between the cGAS/STING pathway and YFV is still unclear. Only very recently, it was shown for the first time that YFV protease complexes behave very differently from those of its other *Flavivirus* cousins [85]. It appears that both strains of YFV NS2B3 (17D and Asibi) are unable to cleave, and thus presumably unable to inhibit hSTING in the overexpression system. In summary, there are still many unknowns about YFV’s modulation of the cGAS/STING pathway, and further studies are required to draw a clearer picture.

### 6.2. Japanese Encephalitis Virus

JEV is another emerging arbovirus that is transmitted by *Culex* mosquitoes in the *Flavivirus* genus [7,135]. As one of the most common encephalitic flaviviruses, JEV infects between 30,000 and 50,000 people every year, with severe cases estimated to occur in 1 in every 250 cases [6,136]. Although the vast majority of infections are asymptomatic, those who do develop encephalitis (usually children) face significant morbidity and mortality risks. It is estimated that one in four symptomatic cases is fatal [6].

Compared with the flaviviruses mentioned earlier in the review, we still understand very little about how JEV interacts with the cGAS/STING pathway. Recently, using a murine model, a group reported upregulation of mSTING (as well as other proinflammatory response markers) both in vivo and in vitro during early JEV infection [76]. They also suggested that mSTING, likely working in tandem with RIG-I, can suppress JEV replication in mouse cells.

In an overexpression system using human cells, the latest evidence on the involvement of JEV viral proteins in the cGAS/STING pathway comes from the report of the Ploss group, showing that JEV NS2B3 behaves somewhere in the middle between ZIKV/DENV/WNV NS2B3 and YFV NS2B3 in its ability to cleave hSTING [85]. Similar to DENV/WNV, JEV NS2B3 protease complex seems to cleave hSTING at the newly proposed cleavage site, but not mSTING. Surprisingly, it failed to cleave the recombinant hSTING when previously proposed cleavage site R95/G96 on hSTING was replaced with that of the mouse sequence, thus behaving differently from ZIKV/DENV/WNV protease constructs in the same experiment. Further exploration will provide us with a better idea of the interaction between JEV viral proteins and the cGAS/STING pathway.

### 6.3. Duck Tembusu Virus

Although the focus of this review is on the mammalian cytosolic DNA sensory system and the flaviviral strategies on regulation and modulation of those pathways, the authors believe it is noteworthy to mention some elucidating new research coming from the field of avian flavivirus (duck Tembusu virus, or DTMUV) [88,89,137].

After the newly emerged avian flavivirus, DTMUV, was identified and isolated in Southeast China, a group of researchers developed a reverse genetics system in ducks that mimics what has been done in mammalian systems [137]. Using this molecular system, they have discovered that the DTMUV protease complex NS2B3 can block duck IFN-I production by inhibiting IFN-I transcription mediated by duck RIG-I, MDA5, MAVS, and STING [89]. Furthermore, they found that DTMUV NS2B3 cleaves duck STING (duSTING) through an NS2B dependent manner and that the binding of NS2B with duSTING distal tail (residue 221–225) is a prerequisite for the subsequent cleaving by NS2B3 complex. Moreover, in their latest work, they discovered that DTMUV NS2A competes with duck TBK1 (duTBK1) to bind with duSTING, thus disrupting duSTING dimerization and inhibiting downstream IFN production [88]. The work they performed using an avian model will undoubtedly inform the work we do in mammalian systems in the future and could potentially provide evidence for a conserved pan-flavivirus strategy of modulating host innate immune space by targeting the cGAS/STING pathway [138,139].

## 7. Perspectives

It remains elusive why a whole genus of single-stranded positive-sensed RNA viruses has evolved a seemingly conserved mechanism targeting an important, mostly cytosolic DNA sensing system. This is also the case for some other RNA viruses, including coronaviruses (SARS-CoV), that are capable of targeting STING [77,140,141,142].

If a DNA virus evolves mechanisms to co-opt, for example, the RIG-I/MAVS pathway, it could be reasoned that DNA viruses have RNA forms as an intermediary step during active infection and that could serve as ligands for the host cytosolic RNA sensing mechanism and alert the cell to mount an antiviral response [143]. Nevertheless, none of the flaviviruses mentioned are known to have a DNA intermediary phase during infection. If the cGAS/STING pathway is triggered during flaviviruses replication, what could serve as the danger signal?

We offered the hypothesis that host mtDNA leakage can occur during DENV replication, and the resultant misplaced mtDNA in the cytosol can serve as ligands that alert the cytosolic innate immune DNA sensors [82,104]. This hypothesis was based on the previous observation that DENV NS4B can disrupt the integrity of mitochondria and dampen innate immune signaling [144]. After using transduced cGAS cells and cGAS pulldown assays, our group provided evidence that mtDNA can be readily detected by cGAS during DENV infection [82]. Together with the recent report that IL-lβ is responsible for inducing the release of mtDNA during DENV infection and subsequent innate immune response activation, mtDNA’s role in acting as the host ligand for cytosolic DNA sensors is further confirmed [145,146]. Nevertheless, is mtDNA the only source of agonist inducing cGAS/STING response during *Flavivirus* infection?

To our knowledge, a comprehensive screening followed by functional validation of putative cGAS ligands of either viral or host origin under viral infection conditions has not yet been done, even when new evidence of host nucleic acid elements modulating and regulating immune responses is increasingly surfacing in recent years [147,148,149,150,151,152,153,154]. One particular field that we propose would broaden our understanding of innate immunity is at the interdisciplinary space between host innate immune sensing and endogenous retroelements (EREs). Although these elements consist of a large part of our genome, we know remarkably little about their potential regulations and functions, especially during viral infections. Growing evidence suggests that viral infections can impact ERE expression levels, and some non-retroviral RNA viruses can even be reverse-transcribed by the host EREs under certain conditions [149,152,154,155,156].

In particular, EREs derived from non-retroviral RNA viruses in insect systems have been actively studied in recent years and are found to provide partial protection against parent viruses. The Saleh group has reported this phenomenon in the form of finding reverse-transcribed viral DNA (from RNA of insect flaviviruses) capable of inducing virus-specific siRNA responses amplification in a range of insect cells [157,158,159,160]. Even when more studies are warranted before a definitive antiviral or proviral role can be ascribed to EREs in mammalian systems during infection, we hypothesize that future studies may show more host nucleic acids can act as agonists for the cGAS/STING pathway during RNA virus infection. This would not only serve to explain further the seemingly paradoxical conserved strategy in silencing the cytosolic DNA sensing pathway by flaviviruses, but also open up a new direction for cGAS/STING research in general.

## Figures and Tables

**Table 1:** Mechanisms of known cyclic GAMP synthase (cGAS)/stimulator of interferon genes (STING) antagonism by flaviviruses. NS, nonstructural; DENV, dengue virus; ZIKV, Zika virus; WNV, West Nile virus; YFV, yellow fever virus; JEV, Japanese encephalitis virus; IFN, interferon; TBK1, TANK-binding kinase 1.

Genus	Species	Protein	Mechanism of cGAS/STING Antagonism	References
*Flavivirus*	DENV	NS2B	Targets cGAS for degradation through an autophagy–lysosome-dependent mechanism.	[82]
		NS2B3	Proteolytically degrades human STING, but not mouse STING.	[10,11,83,84,85,86]
	ZIKV	NS1	Indirectly degrades cGAS via the enhanced stabilization of caspase-1.	[87]
		NS2B3	Proteolytically degrades human STING, but not mouse STING.	[85]
	WNV	NS2B3	Proteolytically degrades human STING, but not mouse STING.	[85]
	YFV	NS4B	Unknown	[19]
	JEV	NS2B3	Proteolytically degrades human STING, but not mouse STING.	[85]
	DTMUV *	NS2A	Competes with duck TBK1 to bind with duck STING, thus disrupting its dimerization and inhibiting downstream IFN production.	[88]
		NS2B3	Proteolytically degrades duck STING through an NS2B dependent manner with the binding of NS2B with duck STING being required for cleaving	[89]

* Duck Tembusu virus (DTMUV) is an avian flavivirus.
